# Construction and Enhanced Efficiency of Bi_2_MoO_6_/ZnO Compo-Sites for Visible-Light-Driven Photocatalytic Performance

**DOI:** 10.3390/nano13010214

**Published:** 2023-01-03

**Authors:** Liyun Yan, Jiahui Tang, Qing-an Qiao, Honglan Cai, Yuqi Dong, Juan Jin, Yanbin Xu, Hongwei Gao

**Affiliations:** 1 School of Chemistry and Materials Science, Ludong University, Yantai 264025, China; 2 School of Life Science, Ludong University, Yantai 264025, China

**Keywords:** Bi_2_MoO_6_/ZnO nanocomposites, photocatalyst, degradation

## Abstract

Bi_2_MoO_6_ was one of the important bismuth-based semiconductors with a narrow bandgap, and has been widely used in selective oxidation catalysts, supercapacitors, and energy-storage devices. A series of Bi_2_MoO_6_/ZnO composite photocatalysts with different mass ratios were synthesized by the hydrothermal method. The synthesized samples were characterized by XRD, PL, UV-Vis, SEM, TEM, XPS, and BET analysis techniques. Under visible light conditions, Methylene blue (MB) was used as the target degradation product to evaluate its photocatalytic performance. The results showed that the degradation rate constant of Bi_2_MoO_6_/ZnO (0.4-BZO) was about twice that of the traditional photocatalysis of ZnO. The Bi_2_MoO_6_/ZnO composite catalyst maintained stable performance after four consecutive runs. The high photocatalytic activity of Bi_2_MoO_6_/ZnO was attributed to the efficient electron transport of the heterojunction, which accelerates the separation of electron-hole pairs and reduces the probability of carrier recombination near the Bi_2_MoO_6_/ZnO heterojunction. Bi_2_MoO_6_/ZnO nanocomposites have potential applications in the field of photodegradation.

## 1. Introduction

With the rapid development of the economy and the continuous improvement of people’s living standards, the resulting environmental pollution problems have become increasingly serious, especially the production of organic pollutants. Among various harmful pollutants, dyes such as rhodamine B (RhB) and methylene blue (MB) were widely used in the dyeing of silk, wool, cotton, nylon, etc. [1,2,3]. These dyes were discharged into wastewater and posed a hazard to living and aquatic species as they were not biodegradable.

Among all pollutant treatment methods, such as adsorption, sedimentation, filtration and photocatalysis, the most effective treatment method is photocatalytic degradation [4,5,6,7]. The commonly used photocatalytic degradation methods include chemical catalysis, biocatalysis, etc. Some inorganic-bio hybrid photocatalyst systems, such as CuS-functionalized cellulose-based aerogel [8,9], can degrade dyes to some extent as well. The disadvantages lay in the fact that the photocatalytic efficiency is low, and some catalysts cannot be recovered or reused. Some metal-oxide based catalysts (including TiO_2_, ZnO, Fe_2_O_3_, etc. [10,11,12]) have low utilization rates under the visible light and low photo-generated charge separation efficiency, which limits their practical application.

At present, semiconductor photocatalysis is widely studied because of low cost and high efficiency in the field of environmental remediation and solar energy conversion. The design and construction of highly active semiconductors were still the research focus of photocatalytic [13,14,15,16,17]. Among them, ZnO was widely investigated due to its low cost, non-toxicity, environmental sustainability, and abundant resources. However, there were still some disadvantages that limit its performance in practical applications, especially, the wide intrinsic bandgap (3.2 eV) [18,19]. As a result, ZnO could only absorb the ultraviolet (UV) light, which was equivalent to 4% of the solar spectrum and greatly reduced the utilization of visible light [20]. 

An ideal photocatalyst should possess narrow band gap energy, low cost, good stability, and high visible light efficiency. Bi_2_MoO_6_ was one of the important bismuth-based semiconductors and had been widely used in selective oxidation catalysts, supercapacitors and energy-storage devices [21,22,23]. Recent studies have shown that Bi_2_MoO_6_ also possesses excellent visible-light catalytic activity with a narrow bandgap (2.5–2.8 eV) and a clear photoresponse to visible light [12,24,25,26,27], which can be used to split water and degrade organic pollutants [28,29,30]. Therefore, Bi_2_MoO_6_ can be used as an excellent modifier to enhance the photocatalytic performance of ZnO.

In recent years, there have been some reports on Bi_2_MoO_6_/ZnO photocatalysts. A novel spherical Bi_2_MoO_6_/ZnO composite catalyst was synthesized by solvothermal method [31].

The photocatalytic degradation efficiencies of the synthesized catalysts for dyes such as methyl orange (MO), rhodamine B (RhB), and methylene blue (MB) were measured under visible light irradiation. The results showed that the synthesized Bi_2_MoO_6_/ZnO catalysts were more efficient than the bare catalysts (Bi_2_MoO_6_ and ZnO) and showed better degradation efficiency. Bi_2_MoO_6_/ZnO hetero-nanosheet array films were synthesized by a combination of hydrothermal reaction and in-situ electrodeposition process [32]. As photoanode, Bi_2_MoO_6_/ZnO heterojunction films exhibited better performance than pure Bi_2_MoO_6_ and ZnO nanosheet arrays under visible light. Another Bi_2_MoO_6_/ZnO composite synthesized by hydrothermal method showed stronger photocatalytic activity by photodecomposing MO under sunlight [33]. To construct Bi_2_MoO_6_/ZnO heterojunction photocatalysts, a simple two-step solvothermal method was carried out [34]. Under visible light irradiation, Bi_2_MoO_6_/ZnO showed higher photocatalytic activity than single ZnO or Bi_2_MoO_6_. However, some questions were still unresolved in these studies, such as long reaction time length (20 h–24 h), long photocatalysis time (3–6 h), and the irradiation with invisible light during degradation, which made the prepared catalysts less practical in real usage. Consequently, the preparation method, structure, and properties of Bi_2_MoO_6_/ZnO composites needed to be further explored. 

Methylene blue (MB) was selected as the target pollutant in this work, because it was found that methylene blue had a wider application range compared with other dyes such as rhodamine B. It can not only be used as a dye, but also has widely application on electrochemical measurement [35], construction of electrochemical sensors [36] and modification of electrochemical reactions on electrodes. Moreover, the application of MB in medical field is more noticeable, for example, MB is used to treat septic shock [37], fluoroacetate poisoning [38], and tumor [39]. Due to the wide range of usages, it is necessary to find an efficient catalyst for the effective removal of MB in the wasted water.

In this work, a new preparation method with short time-consuming and good photocatalytic effect was designed. High temperature calcination was used to prepare Bi_2_MoO_6_/ZnO. Then, a series of Bi_2_MoO_6_/ZnO composite photocatalysts were prepared by loading different contents of BMO onto ZnO via solvothermal method. Taking MB as the target pollutant, the photocatalytic degradation performance of Bi_2_MoO_6_/ZnO photocatalyst was investigated. The photocatalytic activity was significantly enhanced under the synergistic effect of the two steps above.

## 2. Materials and Methods

### 2.1. Chemicals

Bi(NO_3_)_3_·5H_2_O, Na_2_MoO_4_·2H_2_O and Zn(CH_3_COO)_2_·6H_2_O chemicals were purchased from Aladdin. Ethylene glycol and anhydrous ethanol were purchased from Sinopharm Chemical Reagent Ltd. Corp. Shanghai, China The materials were analytical grade (99%) and were used directly without any purification. Methylene blue (MB) was purchased from S.D Fine Chemicals. Deionized water was used for all the experiments.

### 2.2. Methods

#### 2.2.1. Synthesis of Hierarchical Flower-like Bi_2_MoO_6_ Hollow Spheres (BMO)

First, 4 mmol Bi(NO_3_)_3_·5H_2_O and 2 mmol Na_2_MoO_4_·2H_2_O were respectively dissolved in 15 mL ethylene glycol, and then 50 mL of ethanol was added while mixing the two solutions. After stirring 30 min, the pale-yellow solution was transferred to a 100 mL reaction kettle and was heated to 180 °C for 12 h. Finally, it was cooled to room temperature and centrifuged to obtain a pale-yellow precipitate. The sample was washed three times with deionized water and absolute ethanol, respectively, and dried under a vacuum at 80 °C for 12 h. After that, it was annealed at 350 °C for 1 h in air with the heating rate at 15 °C min^−1^. Finally, the yellow powder of BMO was obtained.

The reaction conditions for the preparation of BMO were explored. Two reaction temperatures of 160 °C and 180 °C and three react time lengths (12 h, 18 h and 24 h) were selected to explore the photocatalytic effect of methylene blue (MB). After reacting at 180 °C for 12 h, the maximum degradation rate of the obtained BMO to methylene blue reached 74.4% within two hours. After 18 h of reaction at 160 °C, the maximum degradation rate of BMO within two hours is 71.2% (Table 1). By comparing the catalytic performance, 180 °C and 12 h were selected as the reaction conditions. We compared the degradation effects of the samples calcined at high temperature and untreated ones, and found that the degradation rate (after two hours of reaction) was varied at about 10%. According to references [29], the organic residues on the surface of the composite materials could be further removed at high temperature calcination and prevented secondary pollution. The main deference of this method was the shortened react time length which would direct to low energy cost.

#### 2.2.2. Synthesis of ZnO

2.0 g Zn(CH_3_COO)_2_·6H_2_O was dissolved in 80 mL of ethanol. After ultrasonic treatment for 30 min at room temperature and stirring for 30 min, the mixture of white solution was transferred to a 100 mL reaction kettle, and kept 6 h at 180 °C. When cooling to room temperature, the obtained samples were washed alternately with deionized water and ethanol to remove organic residues in the samples, and zinc oxide powder was obtained after drying at 80 °C for 12 h.

#### 2.2.3. Synthesis of Sphere-like Bi_2_MoO_6_/ZnO(BZO)

In a typical synthesis process, the prepared complexes were individually weighed and called X- Bi_2_MoO_6_/ZnO (X-BZO), where “X” was 0.1, 0.2, 0.3, and 0.4, representing the mass content of BMO. BMO and ZnO were added to 70 mL of ethanol and 10 mL of hydrazine hydrate were added into the mixed solution. Following 30 min of sonication, the mixture was transferred to a 100 mL reaction kettle and reacted for 6 h at 90 °C. After cooling to room temperature, the obtained precipitate was filtered and washed alternately with deionized water and ethanol. By drying at 80 °C for 12 h, the spherical BZO composites were obtained by annealing at 350 °C for 1 h in air.

In addition, the reaction time lengths, 2 h and 10 h, were also selected to generate the Bi_2_MoO_6_/ZnO heterojunction with the temperature kept at 90 °C. The further experiments indicated that 6 h was the most favored time length to form a photocatalyst with notable performance (the degradation rates of MB 92%). 

### 2.3. Characterization

The phases of ZnO, BMO and BZO were determined by X-ray diffraction (XRD, MiniFlex600, Rigaku, Tokyo, Japan). The surface topography of the samples was recorded by field emission scanning electron microscope (SEM, HITACHI, SU8010, Tokyo, Japan). The microstructure of the photocatalyst was analysed by transmission electron microscopy (TEM, FEI, Talos F200S, Thermo Fisher Scientific Inc., Waltham, MA, USA) at 200 kV. The specific surface area and pore size distribution analyzer (BET, ASAP2460, Micromeritics, Norcross, GA, USA) were used to measure the adsorption isotherm of the sample with N_2_ as the adsorption medium. The specific surface area of the samples was analyzed by the Brunauer Emmet Teller (BET) method. Before the test, the sample was degassed at 200 °C; under vacuum for 12 h to remove the air and impurities adsorbed in the channel. The photoluminescence spectra (PL, HITACHI, F-7100, Japan) were used to evaluate the carrier recom-bination. The spectral response range of photocatalyst was analysed by UV-Vis diffuse reflectance spectroscopy (DRS, Shimadzu, SolidSpec-3700, Tokyo, Japan), with the wavelength from 240 nm to 800 nm. The energy gap (Eg) of ZnO, BMO and BZO was determined from Tauc plot method [40]. The elemental composition and valence state of the samples were characterized by X-ray photoelectron spectroscopy (XPS, Thermo Fisher Scientific, ESCALAB Xi+, Thermo Fisher Scientific Inc., Waltham, MA, USA). Al Ka ray was used as the excitation source with an operating voltage of 12.5 kV.

### 2.4. Measurement of Photocatalytic Activity

The photocatalytic performance was mainly determined by the removal of organic dyes under simulated visible light. First, 30 mg catalyst was added to 80 mL methylene blue solution. A 300 W xenon arc lamp with filter was used to simulate the visible light, and the solution was stirred with a mechanical stirrer with speed range of 0–1000 rpm. Before irradiation, a dark reaction (no light and only catalyst present) was performed for 30 min to achieve an adsorption-desorption equilibrium. During the photocatalytic reaction, 3 mL of the solution was withdrawn every 10 min and the concentration of MB was measured. According to the Lambert-Beer Law [41], the concentration of light-absorbing substances in a certain concentration range was proportional to the absorbance. Correspondingly, the absorbance of MB was measured at 657 nm by a UV-Vis spectrophotometer in the same way. The photocatalytic activity of the photocatalyst was studied by analyzing the degradation curves of organic dyes.

## 3. Results and Discussion

### 3.1. Subsection

**Figure 1:**
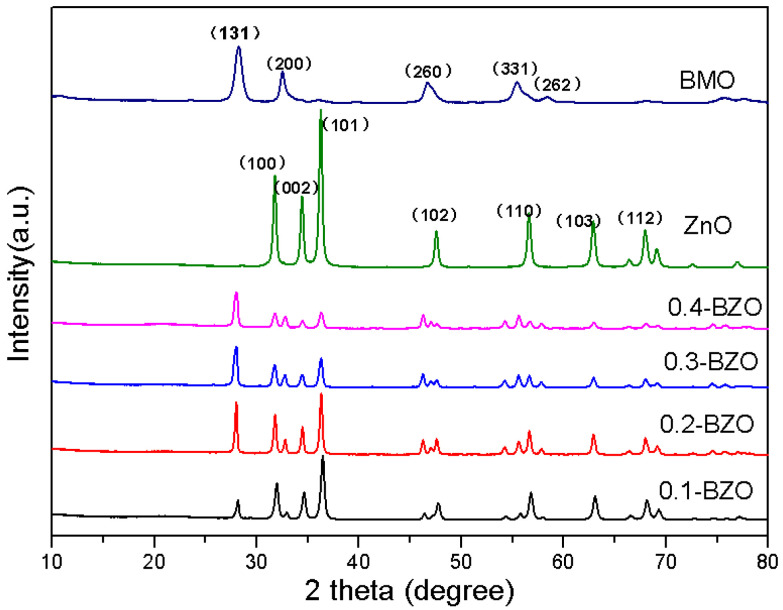
XRD pattern of BMO, ZnO, and BZO.

The XRD patterns of ZnO, BMO, and BZO composites with different mass ratios were shown in Figure 1. The diffraction peaks of pure ZnO were located at 31.8°, 34.4°, 36.2°, 47.5°, 56.6°, 62.9°, which pointed to (1 0 0), (0 0 2), (1 0 1), (1 0 2), (1 1 0), and (1 0 3) of the hexagonal wurtzite structure with space group P63mc (JCPDS 36-1451) [42,43]. For BMO, four peaks located at 28.3°, 32.7°, 46.8°, 55.6°and 58.4° were observed, which were associated with (1 3 1), (2 0 0), (2 6 0), (3 3 1), and (2 6 2) crystal planes (JCPDS 84-0787) [44,45]. Therefore, it can be concluded that the composites were composed of orthogonal BMO nanostructures combined with wurtzite ZnO. With the increase of BMO content, the characteristic peak intensity of ZnO gradually weakened, while the characteristic peak intensity of BMO gradually increased [46]. The peak (2 0 3) at 54.3° corresponded to the impurity Bi_2_O_3_. In addition, there were no traces of other impurity phase, indicating that the prepared sample was of high purity.

### 3.2. BET Surface Area Analysis

**Figure 2:**
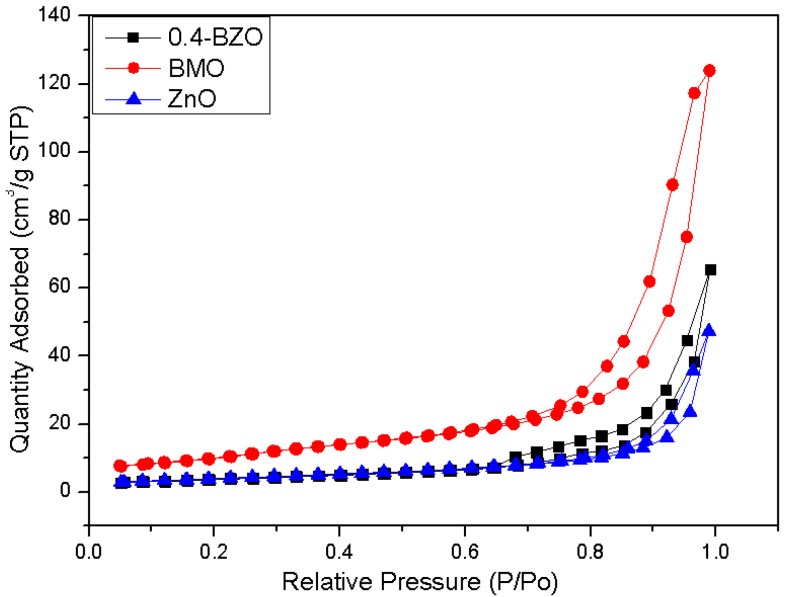
Nitrogen adsorption-desorption isotherms of BMO, ZnO, and BZO catalysts.

The N_2_ adsorption-desorption isotherms of BMO, ZnO, and BZO were shown in Figure 2. Among all the composites, 0.4-BZO had the largest specific surface area (Table 2). The calculated specific surface areas of the BMO, ZnO, and 0.4-BZO catalysts were 38.34, 14.54 and 21.32 m^2^/g, respectively. The specific surface area of BZO was about 1.5 times that of ZnO. All prepared samples exhibited type IV adsorption isotherms with H_3_ hysteresis loops. If not specially pointed out, all results mentioned in the following context were from samples of 0.4-BZO. The high surfaces of BMO and 0.4-BZO were beneficial in high photocatalytic reaction rates.

### 3.3. SEM and TEM Analyses

**Figure 3:**
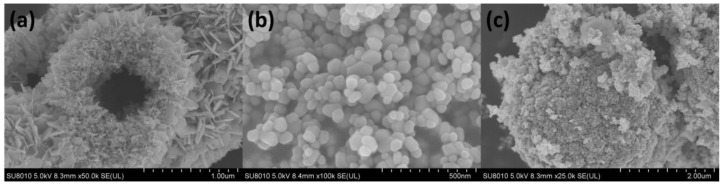
SEM images of BMO (**a**), ZnO (**b**), and BZO (**c**).

The morphologies of ZnO, BMO and BZO were studied by scanning electron microscopy (SEM) (Figure 3). The flower-like hollow microspheres of BMO are shown in Figure 3a. The high surface area of BMO could be attributed to the unique structure. The nanosheets have a relatively smooth surface, which provides a suitable environment for carrying co-photocatalysts. The particle size of BMO microspheres was about 1~2 μm (Figure 3a), and there was a slight agglomeration phenomenon. Pure ZnO was in the form of nanoparticles with a uniform shape of about 50 nm. The presence of ZnO reduced the possibility of BMO agglomeration, and a BZO composite with smaller grain size could finally be obtained. This 3D hierarchical structures can be served as substrates and provide abundant active sites for the adsorption and photocatalytic reactions. 

**Figure 4:**
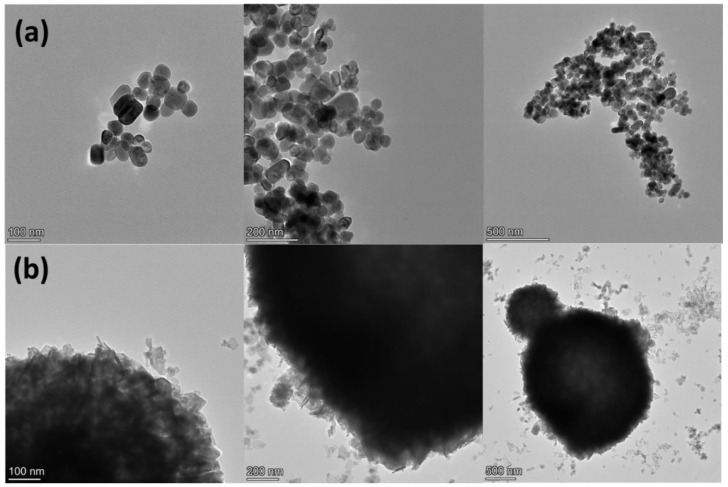

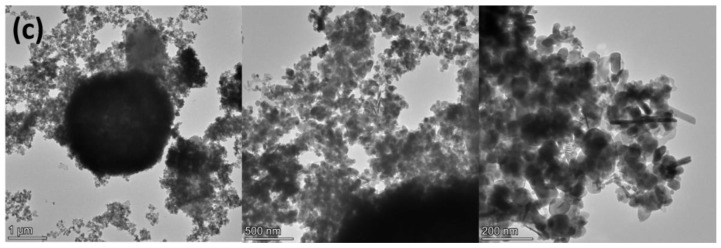
TEM images of ZnO (**a**), BMO (**b**), and BZO (**c**).

The microstructure of ZnO, BMO and BZO were analysed by transmission electron microscopy (TEM), as shown in Figure 4. It could be found that a large number of ZnO nanoparticles were wadded on the surface of the BMO nanospheres (Figure 4a,b), which also confirmed the successful preparation of BZO. The morphologies of BMO microspheres and BZO heterojunctions were similar. As a result, the formation of heterojunctions does not destroy the original morphology of BMO microspheres.

**Figure 5:**
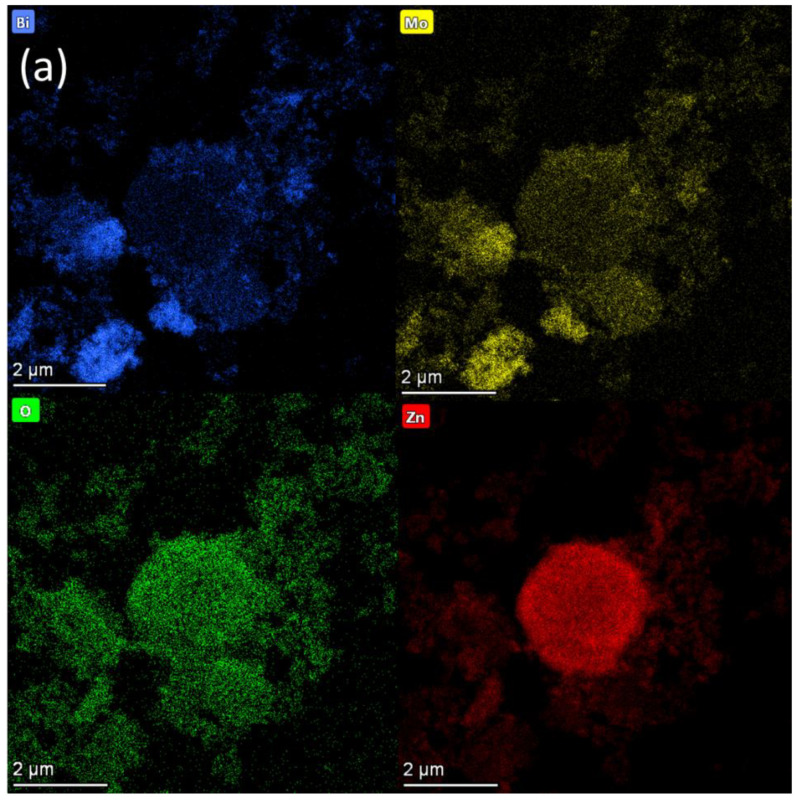

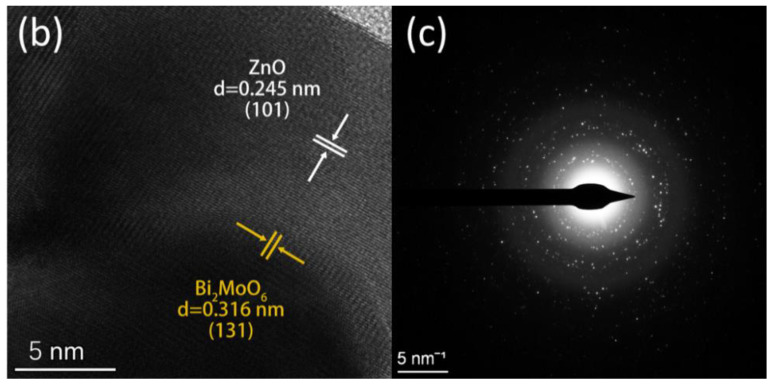
(**a**) TEM image and elemental mapping images of Bi, Mo, O, and Zn of BZO, (**b**) lattice fringes of BZO, and (**c**) SAED diagram of BZO.

The elemental mapping of BZO (Figure 5) confirmed the presence of Bi, Mo, O, and Zn elements in the composite. The XPS data in the following were also consistent with these results. The presence of Zn element can be clearly seen in the energy spectrum, and the ZnO nanoparticles were uniformly distributed on the surface of BMO. The flower-like structure of BMO was basically preserved.

The high-resolution TEM (HRTEM) images (Figure 5b) showed the crystal structure and two sets of lattice fringes with D-spacing of 0.248 nm and 0.315 nm, corresponding to the (101) plane of ZnO and (131) plane of BMO, respectively. The SAED results was shown in Figure 5c. The diffraction ring at the center of the transmission spot was clearly visible, indicating ZnO and Bi_2_MoO6 had excellent polycrystalline properties.

### 3.4. Fluorescence Spectroscopy

**Figure 6:**
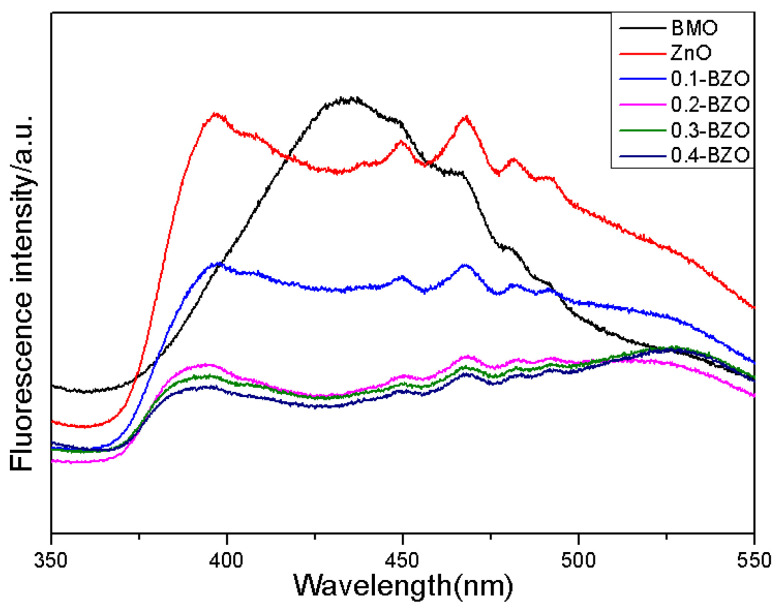
PL spectra of BMO, ZnO and X-BZO at room temperature (the excitation wavelength was 330 nm).

Photoluminescence (PL) was able to be used to evaluate the separation and recombination efficiency of photogenerated electrons and holes in semiconductor photocatalysts. The PL intensity indicated lower recombination rate of photoinduced electron-hole pairs, which would result in more efficient photocatalytic performance [31,47]. The fluorescence intensity comparison of X-BZO with BMO, ZnO is shown in Figure 6. The excitation wavelength was 330 nm. With the increase of BMO content, the fluorescence of X-BZO was gradually weakened and some the peaks were much lower than those of BMO and ZnO. The peak intensity of 0.4-BZO emission was lower than those of other samples. This was attributed to the fact that the addition of BMO makes the surface of the ZnO more exposed, and the number of generated photogenerated carriers would be increased. The intense emission peak at 433 nm for BMO was not observed in 0.4-BZO, suggesting the electron transfer from BMO to ZnO in the heterojunction.

### 3.5. UV–Vis DRS Analyses

**Figure 7:**
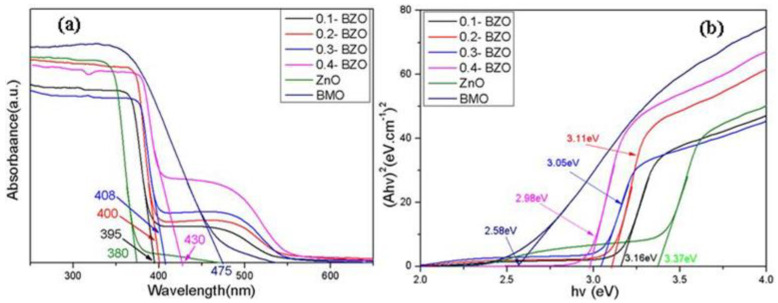
(**a**) The UV–Vis DRS of BMO, ZnO, and X-BZO, and (**b**) variation of (αhν) ^*n*/2^ versus photon energy (hν).

The optical properties of the synthesized samples were investigated by UV-Vis DRS (Figure 7). The absorption edge of X-BZO nanocomposites was shifted towards the visible region due to the addition of BMO. The absorption edge of pristine ZnO was about 380 nm, and that of pure BMO was about 475 nm. With the increase of the proportion of BMO, the absorption wavelength of X-BZO composites was red-shifted, and the edge of the response to light was moved to the visible region. The absorption edge of 0.4-BZO was extended to 445 nm, which showed the best performance. Among all the samples, the X-BZO composite showed significantly improved ability to absorb visible light compared with ZnO. Consequently, more electrons and holes can be generated through the enhanced visible light absorption of the BZO composite.

According to the light absorption of semiconductor photocatalysts, the classical Kubulka–Munk formula [48] is satisfied:

(1) $$\alpha h\upsilon =A{\left(h\upsilon -E_{g}\right)}^{n/2}$$

Among them, α is the absorption coefficient, h and υ are the Plananger constant and incident light frequency, respectively; A and Eg refer to the constant and the band gap energy, respectively; and *n* takes different values according to the type of semiconductor. For the direct transition type, *n* = 1, and for indirect ones, *n* = 4 [49,50]. Both ZnO and BMO belong to direct transition semiconductors, so *n* = 1 was taken [51]. 

The band gap energies of ZnO and BMO and X-BZO composites were obtained via the relationship between (αhν) ^*n*/2^ and energy (hυ) (Equation (1)). The band gap of ZnO was about 3.37 eV (Figure 7b), which was very closer to the reported value of 3.1–3.2 eV [18,19,42,43,52]. The band gap of BMO was about 2.58 eV. Due to the addition of BMO, the band gap value was getting smaller than that of pure ZnO. Among the four X-BZO nanocomposites, 0.4-BZO was the most favorable one with a band gap of 2.98 eV. The synthesized X-BZO extended the absorption response to a certain extent, so the as-prepared composites can be directly used to absorb visible light. It was possible the abundant oxygen vacancies on the monolayer can not only prolong the absorption of light to longer wavelengths, but also narrow the band gap of the catalyst [46].

### 3.6. XPS Analyses

**Figure 8:**
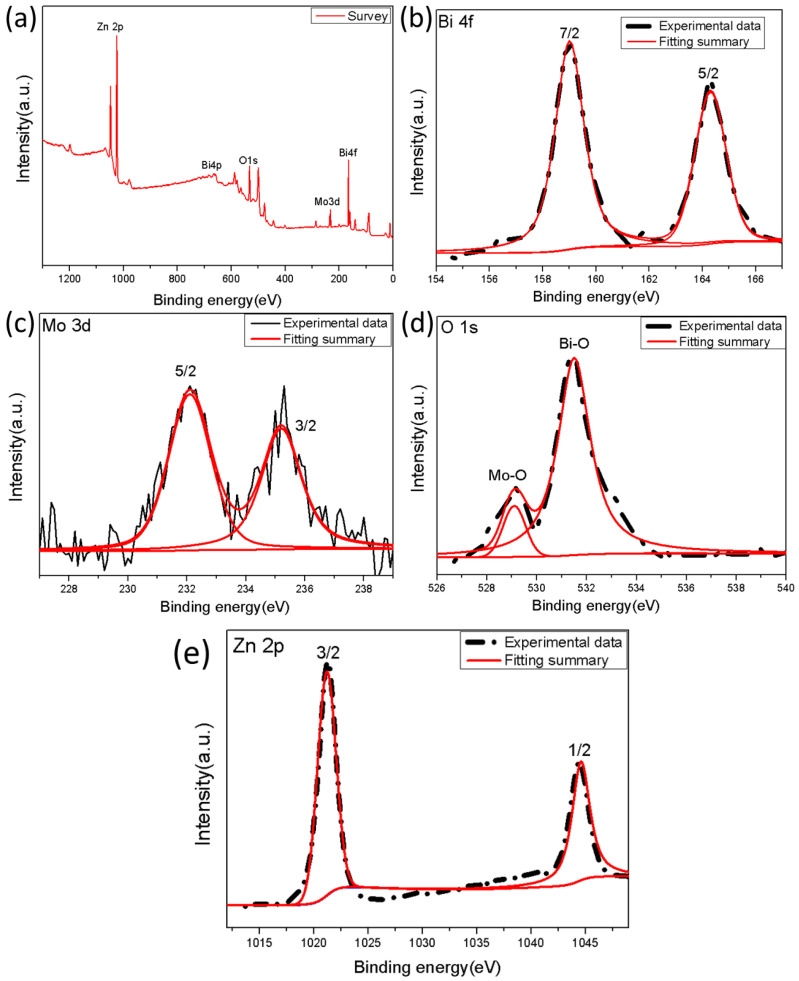
XPS spectra of 0.4-BZO catalyst (**a**) Survey and core level spectra (**b**) Bi 4f, (**c**) Mo 3d, (**d**) O 1s, and (**e**) Zn 2p.

The full spectrum of the photoelectron spectrum of BZO nanoparticles was shown in Figure 8. It can be seen that the sample was composed of four types of elements: Bi, Mo, O and Zn (Figure 8a). No obvious impurity peaks were detected except for the C element which could be attributed to the reference value carried by the instrument. The positions of Bi, Mo, and O peaks were basically consistent with the positions of BZO nanoparticles reported previously [53,54], which indicated that BZO nanomaterials had been successfully synthesized. The two peaks at 159 eV and 164.1 eV in the Figure 8b correspond to Bi 4f_7/2_ and Bi 4f_5/2_, respectively [55]. The two Gaussian peaks with binding energies of 232.3 eV and 235.5 eV correspond to the electron binding energies of Mo 3d_5/2_ and Mo 3d_3/2_(Figure 8c), which were caused by the spin-orbit splitting of electrons, indicating that molybdenum in the form of Mo^6+^ [56]. In addition, the peak shape of O 1 s also exhibited as Gaussian distribution. The peak at 529.2 eV corresponded to the lattice energy between Mo and O, and the peak at 530 eV to the oxygen vacancies between Bi-O metal oxides (Figure 8d) [57]. The peaks associated with binding energies of Zn 2p_3/2_ and 2p_1/2_ were 1021.9 eV and 1045.1 eV respectively [58,59].

### 3.7. Photocatalytic Performance and Mechanism

**Figure 9:**
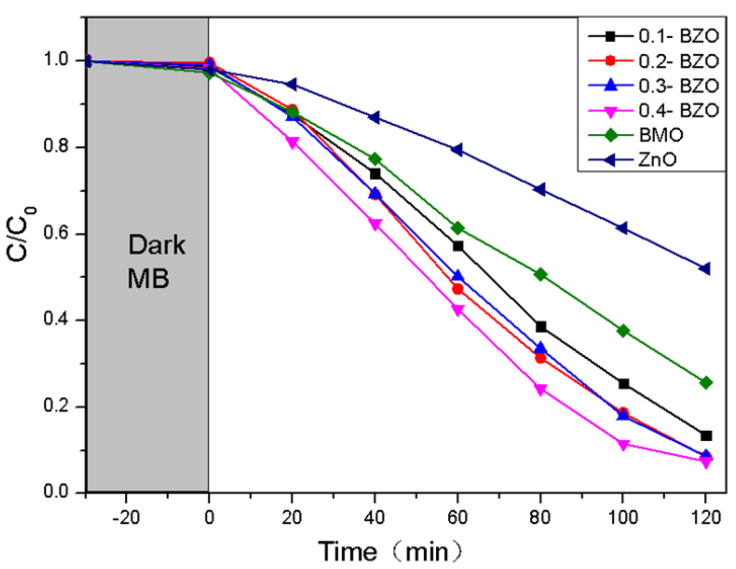
The photocatalytic activity of BMO, ZnO, and X-BZO in 10 mg/L MB dye.

The catalytic performance of the samples under visible light was evaluated with MB as the substrate. After stirring for 30 min under dark conditions, the adsorption–desorption of substrates was equilibrated for all samples (Figure 9). The photocatalytic performance of BMO, ZnO, and BZO composites was evaluated by measuring dye degradation under simulated visible light irradiation. The MB degradation rate was measured as 74%, 48%, 86.5%, 91.3%, 91.4% and 92% at 2 h for BMO, ZnO and four X-BZO complexes. When the content of BMO was increased from 20% to 40%, the catalytic efficiency was not greatly improved. Among the prepared samples, pure ZnO showed the worst photocatalytic effect compared with other samples. When BMO was put in, the dye degradation rate was sharply increased.

Among all the four composites, 0.4-BZO had the best degradation effect, and the degradation rate of MB was reached to 92%. The enhanced photocatalytic performance may be resulted from the narrow band gap of BZO and effective promotion of electron transfer in the heterostructure [31].

The dose for the photocatalyst was smaller in this work (Table 3) compared with other studies [42,60] on photocatalytic degradation. In some cases [31,33,60], a 500 W lamp was used in the reactor for visible irradiation, and they used high power light source compared to this work (300 W xenon lamp). What is more, the photocatalytic time of this work for degradation was shorter than those reported by refs. [31,34,60,61,62]. By comparison, it can be found that a high dye concentration meant a long photocatalytic time or high-power light source [33,60]. If the dye concentration was kept at 10 mg/L, the photocatalytic time or the light source in this work was favorable. Overall, the catalytic efficiency was noticeable higher, which means Bi_2_MoO_6_/ZnO photocatalyst had a broad application prospect in practice. 

**Figure 10:**
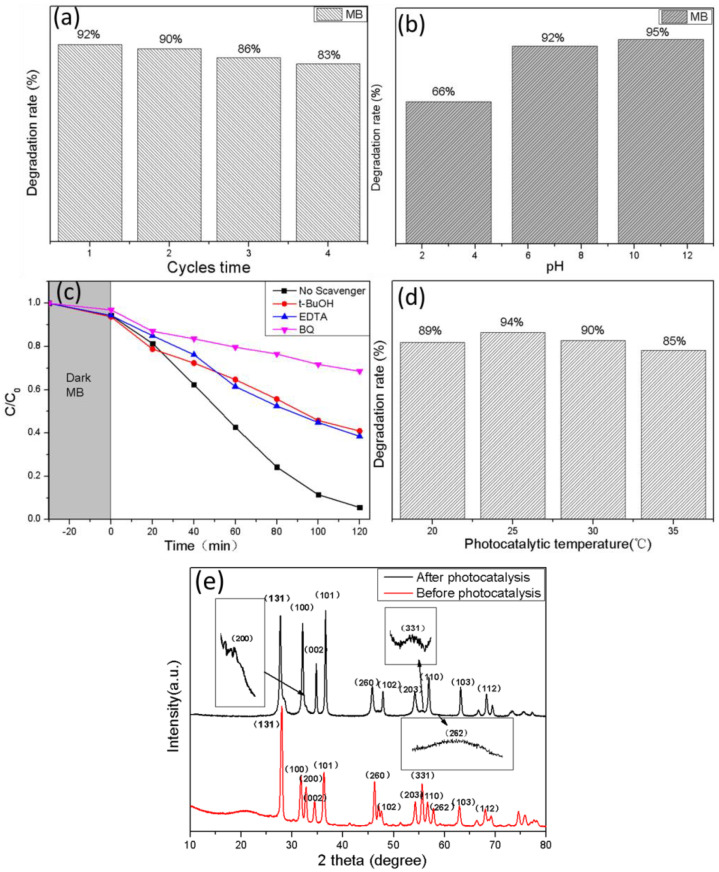
(**a**) Recycling test of photocatalytic MB degradation over BZO catalyst under visible light. (**b**) Degradation rate of BZO at different pH values. (**c**) Photocatalytic activity of BZO for degradation of MB with different quenchers. (**d**) Degradation rate at different temperatures. (**e**) XRD images before and after photocatalysis.

The results of the recycling experiments are shown in Figure 10a; the degradation rate of MB by 0.4-BZO decreased from 92% to 86%, and finally stabilized at around 83%, after four times of recycling. As a result, Bi_2_MoO_6_/ZnO photocatalyst had excellent stability.

The effect of different pH values on the photodegradation of dyes has been explored, eg. photocatalytic efficiency on RhB degradation at pH 5, 7 and 11 [63], the different pH values (pH = 1,4,7,10) on the degradation of RhB [64] and the settling behavior of TiO_2_ at different pH values (pH = 3.60, 5.98 and 9.15) [65]. It could be found that the different pH values represented acidic, neutral, and alkaline conditions for the reaction. Consequently, the photocatalytic degradation under three conditions of pH = 3, 7, and 11 were investigated (Figure 10b). It can be seen that under neutral (pH = 7) and alkaline conditions (pH = 11), the adsorption of dyes by the catalyst were significantly better than that under acidic conditions (pH = 3). This might be caused by the increase of OH^−^ in the reaction environment, which would lead to the increase of ·OH and had a positive effect on the photocatalytic reaction [66]. Likewise, the degradation rate of the catalyst was the worst at pH = 3. Due to the fact that the catalytic efficiency for cases pH = 7 and pH = 11 were similar, pH = 7 was selected economically as the experimental condition because of low energy cost. In order to study the main oxide species in the photocatalytic synergistic degradation of MB by BZO, EDTA, p-benzoquinone (BQ) and tert-butanol (t-BuOH) were used as scavengers to capture holes (h^+^), superoxide radicals (•$O_{2}^{-}$) and hydroxyl radicals (·OH), and the degradation situation without scavenger was used as the control group [67]. All species had significant roles in the photocatalytic degradation process. Compared with other substances, p-benzoquinone played the most significant role in the degradation reaction, which indicated that superoxide radical (•$O_{2}^{-}$) played a major role in the degradation reaction (Figure 10c). The effects of temperature on the degradation of MB were investigated at different temperatures (Figure 10d). One can easily find that the degradation rate at room temperature was the best (94%). As shown in Figure 10e, XRD patterns before and after photocatalysis were analyzed, and it was found that several peaks, such as (200), (331), and (262), widened after photocatalysis. This may be caused by the decrease of the particle size, defects, or internal stress during the photocatalytic degradation [68,69].

**Figure 11:**
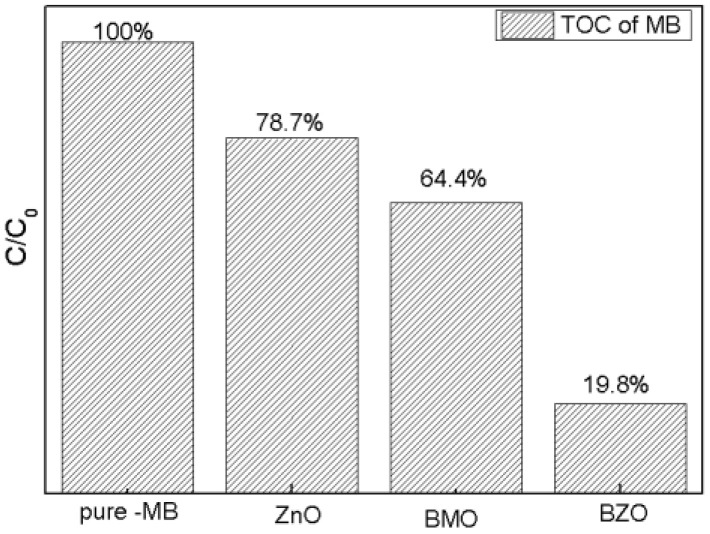
TOC analysis of different systems.

Total organic carbon (TOC) was measured to study the degradation results of BZO compared with ZnO and BMO. The results were shown in Figure 11. It was worth noting that the degradation efficiency of BZO obtained was remarkably higher than that of single ZnO or BMO, which indicated that BZO had a better degradation effect.

**Figure 12:**
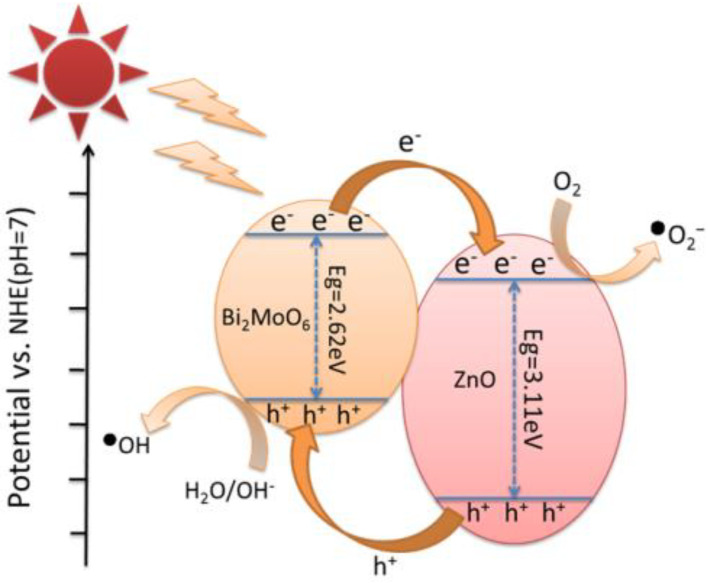
Photocatalytic mechanism of BZOunder visible light.

Based on the abovementioned experimental results, a possible photocatalytic mechanism was speculated (Figure 12). The high photocatalytic activity of BZO was attributed to the efficient electron transport of the heterojunction. Under visible light irradiation, electrons were excited from the valence band (VB) to the conduction band (CB). The excited electrons in the CB of BMO were moved to the CB of ZnO and reacted with dissolved oxygen to form •$O_{2}^{-}$. The holes generated in the VB of ZnO were moved to the VB of BMO and then reacted with the aqueous medium to generate •OH radicals from OH^-^ [26]. The migration of holes from the valence band of ZnO to BMO were built an effective internal electric field that further accelerated the separation of electron-hole pairs and reduced the probability of carrier recombination near the BZO heterojunction. The generated •$O_{2}^{-}$ and •OH were transferred from the BZO to the dye and degrade the harmful substances. There were some previous studies on the role of $O_{2\;}^{-}$ in the degradation of other dyes by Bi_2_(O,S)_3_/Mo(O,S)_2_ nano-composite [70]. It was found that superoxide radical played a major role in the photodegradation of MO. For the prepared Ag/AgBr/NiFe_2_O_4_ photocatalyst [64], the main reactive oxygen species for RhB degradation under visible light was also superoxide radical. Therefore, it could be proposed that $O_{2}^{-}$ played a major role in photocatalysis.

## 4. Conclusions

In conclusion, flower-like BMOs were synthesized by a hydrothermal method under the favored preparation condition of 180 °C, 12 h, and BZO heterojunctions were prepared by physical mixing hydrothermal method on this precursor. ZnO nanoparticles were uniformly dispersed on the flower-like hollow sphere structure. The 0.4-BZO heterojunction had a large specific surface area of 21.32 m^2^/g. Its PL and DRS values were the lowest among the products, which had the highest photocatalytic performance among the as-prepared products. The photocatalytic activity of BZO heterojunctions for MB degradation under visible light was investigated. Compared with pure ZnO and BMO, the photocatalytic performance of 0.4-BZO was significantly improved. The degradation rate of MB by 0.4-BZO was as high as 92%. When the content of BMO was increased from 20% to 40%, the catalytic efficiency was not greatly improved. The catalytic efficiency for different pH values were explored, and it was found that the cases for pH = 7 and pH = 11 were similar. As a result, pH = 7 was selected as the photocatalytic condition economically. In addition, after four cycles of testing, the MB degradation rate remained above 83%, which meant good stability of BZO. The free radical scavenger experiments showed that superoxide radical (•$O_{2}^{-}$) played a major role in the degradation reaction. The excited electrons in the CB of BMO were transferred to the CB of ZnO and reacted with dissolved O_2_ to form •$O_{2}^{-}$, and then moved from the BZO to the dye together with •OH to degrade the harmful substances.

## Figures and Tables

**Table 1:** Preparation conditions of BMO.

Temperature/°C	Reaction Time/h	Photocatalytic Degradation Rate/%
	12	74.4
180	18	73.6
	24	67.7
	12	67.3
160	18	71.2
	24	60

**Table 2:** Specific surface area of BMO, ZnO and BZO.

Sample	BMO	ZnO	0.1-BMO	0.2-BMO	0.3-BMO	0.4-BMO
S_bet_ (m^2^/g)	38.34	14.54	15.95	16.68	18.48	21.32

**Table 3:** Comparison of photocatalytic performance.

Photocatalyst	Organic Dyes	Dosage	Dye Concentration	Photocatalytic Time	Light Source	Efficiency	Ref.
Bi_2_MoO_6_/ZnO	MB	-	10 mg/L	180 min	500 W, Tungsten lamp	91%	[31]
Bi_2_MoO_6_/ZnO	MO	2 mg/mL	20 mg/L	60 min	CHF-XM-500 W	95%	[33]
Bi_2_MoO_6_/ZnO	MO	1 mg/mL	10 mg/L	6 h	300 W xenon lamp	Nearly 100%	[34]
ZnO/GO	MB	0.8 mg/mL	5.0 × 10^−5^ mol/L	60 min	300 W, Xe light	98.1%	[42]
ZnO/γ-Bi_2_MoO_6_	MB	1.5 mg/mL	20 mg/L	240 min	500 W, Xe lamp	89.6%	[60]
Sb_2_O_3_/ZnO	MB	-	10 mg/L	90 min	UV light	71%	[61]
ZnO/Bi_2_MoO_6_	RhB	-	5 mg/L	350 min	300 W Xenon lamp	60%	[62]
Bi_2_MoO_6_/ZnO	MB	0.375 mg/mL	10 mg/L	120 min	300 W xenon arc lamp	92%	This work

## Data Availability

The data presented in this study are available on request from the corresponding author.
